# Selective adsorption of lipopolysaccharide in the complex treatment of patients with severe sepsis

**DOI:** 10.1186/cc12005

**Published:** 2013-03-19

**Authors:** S Rei, I Aleksandrova, V Kiselev, M Ilynskiy, G Berdnikov, L Marchenkova, G Bulava, N Borovkova

**Affiliations:** 1 Hospital Research Institute for Emergency Medicine named after N.V. Sklifosovsky, Moscow, Russia

## Introduction

Severe sepsis and septic shock remain the most serious problem of critical care medicine with a mortality rate of 30 to 55% [1]. Several studies have demonstrated positive effects of selective adsorption of LPS on blood pressure, PaO_2_/FiO_2 _ratio, endotoxin removal and mortality [2,3]. The purpose of the study was to evaluate the efficiency of using the selective adsorption of LPS, Toraymyxin - PMX-F (Toray, Japan) and Alteco^® ^LPS Adsorber (Alteco Medical AB, Sweden), in the complex treatment of patients with severe sepsis.

## Methods

Forty-six patients with Gram-negative sepsis in the postoperative period were enrolled into the study. Toraymyxin - PMX-F was used in the PMX-F group (*n *= 14), while Alteco LPS adsorption was used in the Alteco LPS group (*n *= 32). The clinical characteristics are listed in Table 1. The SOFA score, PaO_2_/FiO_2_, procalcitonin (PCT), C-reactive protein (CRP), endotoxin activity assay (EAA) was noted before, 24 and 48 hours after the selective adsorption of LPS.

**Table 1:** Clinical characteristics of the groups

Characteristic	PMX-F	Alteco LPS
Sex M/F	9/5	17/15
Age (years)	41.6 ± 17.1	42.1 ± 13.5
Shock	6 (42.9%)	13 (40.6%)
APACHE II	21 (17; 25)	21 (16; 24)
SOFA	7 (3.0; 8)	7 (3.5; 11)

## Results

At 48 hours after PMX-F, significantly decreased PCT from 17.5 (5.0; 40.9) to 7.1 (4.8; 13.0) ng/ml, *P *= 0.028, decreased CRP from 180 (133; 286) to 132 (68; 155) mg/l, *P *= 0.015 and SOFA score from 7.0 (3,0; 8.0) to 6.0 (3,0; 7.0), *P *= 0.007. At 24 hours after Alteco LPS, significantly decreased PCT from 8.7 (3.0; 25.9) to 4.8 (2.1; 10.0) ng/ml. The 28-day mortality rate was 14.2% (*n *= 2) in the PMX-F group and 31.3% (*n *= 10) in the Alteco LPS group.

## Conclusion

The use of the LPS-selective adsorption (particularly PMX-F) in patients with severe sepsis leads to improvement of systemic inflammation and organ dysfunction.
